# American Dental Association

**Published:** 1893-03

**Authors:** 


### Reports of Society Meetings.

### AMERICAN DENTAL ASSOCIATION.

(Continued from page 118.)

### *Third Day.—Morning Session.*

The President called for a discussion on the report of Section III., Operative Dentistry, and there being none, the Section was passed.

Section IV., Histology and Microscopy, was called, and, there being no response, was passed temporarily.

### Section V.

### MATERIA MEDICA AND THERAPEUTICS.

Dr. Harlan, chairman of the Section, reports that it has two papers to present,—one by the secretary and one by the chairman. The secretary, Dr. Hunt, then read his report, as follows:

"The past year has been one of activity to the dental therapeutist. Antisepsis and anæsthesia are the two subjects receiving special attention.

"PENTAL.

"Pental, an old compound with a new name, has been recently introduced as a general anæsthetic, and a considerable degree of success has attended its use. Pental is a colorless, highly inflammable, very volatile liquid, soluble in alcohol, ether, and chloroform, but insoluble in water. Unlike sulphuric ether, it is absolutely non-irritant to the mucous membrane of the mouth and respiratory passages, and its exhibition is unattended by nausea, or by a stage of exhilaration and muscular activity. Spasmodic contractions of the muscles of mastication seldom occur, and will readily yield to a further administration of the drug when they do take place. Anæsthesia is produced as rapidly as with nitrous oxide, but the action is more prolonged, and the patient may be kept under the influence for an extent of time sufficient to admit of any of the major surgical operations being performed. The regaining of consciousness is gradual, and the recovery rapid and complete. Full anæsthesia is induced in from three to five minutes, narcosis lasting four or five minutes.

"Pental may be administered in the same manner as ether, but its extreme volatility is better overcome by the use of a modified form of Junker's inhaler, lessening the time of producing narcosis, as well as the amount of the drug used. This latter is an item to be considered, inasmuch as the present price of the compound (six dollars for two pounds eight ounces) renders it rather an expensive article of the materia medica.

"As yet, there have been no alarming symptoms reported from the use of pental on man ; but as eminent a therapeutist as H. C. Wood, M.D., has recently declared, after making a few experiments by inhalation, and by injection into the veins of dogs, that, in his judgment, pental is a dangerous anæsthetic, more so than chloroform, and that, if extensively used, it will produce death by cardiac arrest. Dr. Wood further states that he believes the after-effects of pental on the human being would be disagreeable, a statement directly opposed to the evidence submitted by Dr. Hollander, of Berlin, from whose paper on this subject the major portion of this report is derived.

"Dr. Wood did not produce death from the use of pental, but in one case, under the lethal dose of two grammes, injected into the jugular vein of a dog, the effect on the beast was very alarming, the action of that organ being completely arrested a half-minute before the cessation of respiration.

"In view of these experiments, it would be well to use pental with great caution until the physiological effects of the drug are more definitely determined than they are at present. Further investigation of this subject is now progressing.

"CHLORIDE OF ETHYL.

"Chloride of ethyl has proven itself a local obtundent of considerable worth. For minor surgical operations, such as opening abscesses, lancing boils, extracting teeth, etc., it is admirably adapted, and it also has an appreciable value as an obtundent of sensitive dentine, but in this latter capacity must be used with sufficient caution to protect the pulp from deleterious effects.

"Chloride of ethyl is, at present, put up in ten-gramme flasks. Tubes of one-half the size would be amply large for most of the cases in which we wish to employ the drug. Five grammes are sufficient for the extraction of from one to four teeth, or for any other short minor surgical operation. The extreme volatility of the ethyl renders difficult any attempt to save a portion of the liquid for future use after the neck of the flask has been broken, especially in offices where extracting teeth and lancing abscesses are not daily occurrences. According to recent experiments by Dr. H. C. Wood, chloride of ethyl is a dangerous agent, unsafe and difficult of administration, owing to its extreme fugaciousness.

"ARISTOL.

"Aristol, a combination of iodine and thymol, has now been before the dental profession about eighteen months. To Dr. W. H. Whitslar, of Cleveland, Ohio, is due the credit of first calling our attention to the value of this new antiseptic. Aristol is designed to take the place of iodoform, iodol, and sozo-iodol. It is almost inodorous, and is perfectly non-irritant. It is in the form of a reddish- or salmon-colored powder, insoluble in water, slightly soluble in alcohol, and freely soluble in ether and chloroform. The chemical combination is readily broken up, liberating iodine and thymol, and to this feature of its composition is due its antiseptic action. Aristol may be used in connection with chloro-percha for root-canal fillings, and, dissolved in chloroform in the proportion of sixty grains to the half-ounce, it makes a dressing that may be used in root-canals, or as a protective and antiseptic over excoriations of the skin. The powder may be dusted in gangrenous pulps with a fine brush. An excellent root-canal dressing may be made by rubbing up a grain of aristol in ten or twelve minims of one of the essential oils, to which may be added two or three drops of ether or chloroform to facilitate the process.

"Small rods composed of cocoa-butter and aristol, in the proportion of ten to one, are recommended for use in fistulous tracts. Aristol should be kept in well-soldered, dark-colored bottles, and bought in small quantities to insure freshness, as it deteriorates on exposure to light and air.

"ARGENTI NITRAS (SILVER NITRATE).

"The use of silver nitrate for the arrest of decay, although not a new feature in dental therapeutics, has recently received additional attention, owing to the experiments made and results achieved by Dr. E. A. Stebbins, of Shelburne Falls, Massachusetts. In the deciduous teeth, or in the adult molars, where imperfection in the formation of enamel has resulted in superficial decay, and especially in those long, narrow, sensitive cervical cavities so frequently met with, it may be advantageously employed. In cases of approximal decay in the anterior deciduous teeth, the Arthur method of V-shaped separation may be used to produce a saucer-shaped cavity, and after cutting away the comparatively good tooth-substance, a thorough application of the silver nitrate will prevent a recurrence of caries. A serious and ever-present objection to the use of silver nitrate is the blackness of the cavity after its application; but in the deciduous teeth this drawback is more than counterbalanced by its ease of application and the subsequent freedom from decay.

"BICHLORIDE OF MERCURY.

"The following is an abstract from a thesis entitled 'Corrosive Sublimate as a Germicide,' by Charles T. McClintock, A.M., assistant to the Professor of Hygiene in the University of Michigan. The conclusions arrived at are so totally opposed to all past teachings that it is offered here without comment:

"'In 1881 Koch recommended corrosive sublimate as the most efficient of all known substances for disinfecting purposes. Since that time it has been universally used. After making some hundreds of experiments, the author finds that Koch, and those who have confirmed his work, base their conclusions on faulty experiments, the most important of which was the failure to notice that the sublimate formed with the gelatinous coat of bacteria a compound insoluble in water, but soluble in salines, and readily removed by the blood. When bacteria treated with sublimate were transferred to gelatin or agar, the capsule of mercury prevented the growth of the germ, and the false conclusion was drawn that it was dead. In the author's experiments this capsule of mercury was removed by precipitation with hydrogen sulphide.

"'While Koch states that the bacteria are killed in a few minutes by solutions of sublimate, 1 to 1000, experiments show that bacteria such as staphylococcus pyogenes aureus may grow after having been in the 1 to 1000 solution ninety-three hours, 1 to 100 eleven hours, and a saturated solution one hour. Bacillus subtilis grew after lying in 1 to 100 forty-one hours, saturated solution eighty-five minutes. Typhoid germs in 1 to 1000 one hour, germs in fæces after twenty-four hours in saturated solution. Several experiments comparing strong vinegar with 1 to 1000 sublimate show that they have about the same germicidal powers.

"'The experiments also indicate that a larger number of bacteria in a given culture are comparatively easily killed, and that the ratio of those killed by a germicide to those that survive is of no value. The conclusions drawn from the experiments are:

"'1. That the high rank heretofore given corrosive sublimate as a germicide is without warrant, and was based on faulty experiments.

"'2. Different cultures of the same germ may vary largely in their resistance to germicidal agents.

"'3. Corrosive sublimate forms with cellulose, with albuminous bodies, and with some parts of bacteria a compound that cannot be removed without washing. When acting on a germ, it forms a capsule around a germ, which protects it from the further action of the germicide, and in turn prevents the growth of the germ, unless removed; but this capsule may be removed by the salines, as in the blood. The action of sublimate on bacteria is probably closely analogous to that of alcohol, etc.

"'4. The presence of a gelatinous substance in many, if not all, bacteria has not received due attention from writers on this subject.

"'5. In albuminous fluids, and practically all disinfection has to do with such, corrosive sublimate of any strength whatever is not a reliable germicide.

"'6. While sublimate has no great germicidal powers, it does not follow that it may not be a valuable disinfectant. This point remains to be proved.

"It may be well in this connection to call attention to the fact that the author of the foregoing fails to mention that Laplace long since wrote of the coagulating influence of bichloride on albumen, and demonstrated that the use of a solution acidulated with ether, hydrochloric, tartaric, or even carbolic acid, was not followed by that result.

Dr. Harlan then read the following paper, entitled

### EUROPHEN.

One of the more recent products of the present activity in the world of research is europhen.

Dr. Harlan quoted from F. Goldman the process of producing europhen. Isobutylcresol is first obtained by the action of isobutylalcohol upon *o*-cresol in the presence of chloride of zinc at an elevated temperature. This in aqueous solution is acted upon by iodine in iodide of potassium. Instead of using iodine as such, iodine compounds may be employed, from which the iodine is liberated by oxidizing agents like chloride of lime. The iodine compound arising from this reaction is isobutylorthocresoliodide, or europhen, an amorphous, yellow precipitate, which, separated by filtration and cleansed by washing, is dried at an ordinary temperature, with the exclusion of light.

By analysis, the percentage of iodine (according to a series of determinations made under similar conditions by the method of Carius) was found to average 27.6, which corresponds pretty accurately to the proportion of one atom of iodine to two molecules of isobutylcresol.

Europhen is a yellow, amorphous powder, resinous to the touch, which adheres tenaciously to the mucous membrane, to wounds, and to the unbroken skin. It has a specific, aromatic odor, which reminds us a little of one of its components, *o*-cresol; perhaps, also, of saffron. The iodized phenols, too, which are produced from the above-named alcohols, differ little from europhen as to their aroma. The odor becomes apparent only when europhen is used as such,—*i.e.*, as an unmixed powder. It vanishes almost completely in a mixture or solution.

In reference to solubility europhen has extraordinary advantages. While it is insoluble in water and glycerin, it is easily soluble in alcohol, ether, chloroform, and such compounds of these as collodion and traumaticine, or liquid gutta-percha. As with aristol, the easy solubility of this iodine product in fatty oils is also worthy of notice. We may by rubbing at a low temperature readily produce a twenty-five-per-cent. solution in olive oil. This should be filtered. In alcohol (thirty-per-cent.) it is easily soluble, as also in an equal weight of ether or chloroform. A small precipitate, which forms not only in the oily solution, but also in alcohol, ether, and chloroform, consists of an organic iodine compound, soluble in water and insoluble in the above-named menstrua. A filtered solution in oil remains clear for a long time. It has been proved that neither a ten-per-cent. nor a twenty-five-per-cent. solution gives a precipitate after being kept for more than three months.

If a filtered solution of europhen in oil is shaken with water, the formation of this iodine compound can be seen for days, as with the undissolved powder. What has been said of the vegetable fat (as olive oil) applies equally to ointments prepared with animal or mineral fats (adeps, lanolin, vaselin).

The supposition appears justifiable that europhen in contact with water (wound secretions) gives off slowly small quantities of iodine, which are immediately combined again, and thereby form the soluble compound. Perhaps the chief part of the action of europhen is to be ascribed to this formation in the nascent state.

The intermediate occurrence of free iodine can be shown by bringing europhen, which has become free from iodine by long digesting with starch solution, after washing and while yet moist, into intimate contact with starch solution (not the starch solution of the German Pharmacopœia, but resulting from boiling amylum with water), and leaving the mixture upon the filter itself till the moisture has evaporated. By this means the residue becomes blue, as also does the filter. If we wash it anew with water, the filtrate also will be turned blue. That part of the filter which was most exposed to the light shows a correspondingly stronger iodine blue, in consequence of the affinity of iodine for starch, before the formation of the soluble iodine compound.

There must, therefore, be present in europhen extremely small amounts of free iodine, which form during the drying, and cannot be removed from the product. The percentage is so small that the trace of iodine need not be considered. From ten examinations I compute it on the average at 0.03 per cent.

Europhen is an easily decomposable substance. Therefore, in applying it, certain precautions must be taken. The solution in alcohol slowly gives off small amounts of iodine, which increase with time. In ether the liberation is somewhat greater. If europhen is mixed with fats combined with starch, decomposition occurs, which increases hourly, until finally the paste has assumed a blue-black color. This occurs more rapidly if a few drops of water are added. Therefore all mixtures which contain starch, as, for example, zinc, amylum paste, are to be avoided. On the other hand, europhen conducts itself quite indifferently to talcum, and the practitioner can avail himself of this substance in cases in which a paste of thick consistency is indicated. Mixtures with metallic oxides (zinc oxide, mercuric oxide) and the salts of mercury are to be avoided. On the other hand, simple mixtures with fats, such as vaseline or lanolin, are very durable. Mixtures with lanolin are especially to be recommended, as this substance takes up a large amount of water and favors a continuous formation of the soluble iodine compound.

The solution in alcohol-ether is most used for the preparation of europhen bandages. In its preparation rapid manipulation is a necessity on account of the already mentioned easy liberation of iodine, the amount of which increases with the time employed. For the preparation of vaginal and other suppositories, it is advantageous to dissolve the europhen in some almond oil, and then rub with oil of cocoa. The solution in alcohol or ether, with a partial or complete evaporation of the menstruum, is, on the contrary, unreliable. All solutions should be prepared at a low temperature. Warming causes a transposition and decomposition of the preparation. Europhen must be protected from the light and kept in a dry place.

Finally, we will add another observation, which concerns the volume of europhen as compared to other substitutes for iodoform. If we regard the specific gravity of europhen as 5, that of sozo-iodol is 7.5, of iodol 10, of iodoform 26,—*i.e.*, europhen is five times lighter than iodoform, and five times as large a wound surface can be covered with it as with the same weight of iodoform,—a fact of no small importance. Then the fine iodoform powder cakes very easily, a disadvantage which does not occur with europhen. If europhen is to be a substitute for iodoform, it must possess, in the first degree, the proper anti-bacterial properties. The very thorough investigation by Siebel in reference to this has proved that it is able to exert the same influence as iodoform upon bacteria in cultures on artificial media. Neither europhen nor iodoform destroys the micro-organisms, but simply makes the medium unsuitable for the growth of the bacteria, and thereby hinders the formation of the products of decomposition. It was also established with certainty by further trials that its influence upon the growth of bacteria does not depend upon a mechanical, but upon a chemical action.

The conduct of europhen towards bacteria outside of the organism harmonizes with the results achieved in therapy, and these correspond completely to the claims as to its effects which have been hitherto made for iodoform.

Furthermore, europhen not infrequently proves efficacious in cases where iodoform has been refractory. Europhen has also the advantage over iodoform of a relative freedom from odor and the absence of toxic effects. Its low specific gravity is also worthy of mention. This renders it possible to insufflate as large a wound surface with one part of europhen as with five parts of iodoform, and with equal effect.

Europhen is one of the lightest of the substances forming its group, having two-thirds of the specific gravity of sozo-iodol, half that of iodol, and less than a fifth of that of iodoform, so that a given surface would require five times as much iodoform as europhen to cover it. The latter has also the additional advantage of not caking so easily as the former. Ointments and solutions of europhen must be prepared cold, and solutions require filtration, as an insoluble iodine compound tends to form, which sometimes causes them to assume a gelatinous consistency. Europhen is not poisonous. Half a drachm or more can be given to a dog with impunity. In the human subject, fifteen grains produce no disagreeable symptoms, except perhaps a slight feeling of weight in the stomach.

Dr. Eichhoff has prescribed it with great advantage in several venereal and syphilitic cases, obtaining very satisfactory results, in both soft and hard chancres, in mucous patches, and in tertiary ulceration, by means of one or two per cent. ointment. Excellent results were obtained by the application of europhen to simple ulcers, either in the form of powder or in that of ointment. In general, it was found that europhen acts only when brought in contact with secreting surfaces, whereby it is decomposed and iodine liberated. As exposure to light decomposes europhen, as it does aristol, it is important that these substances should be kept in dark-glass bottles. Bacteriological experiments have been carried out by Dr. Siebel, who finds that europhen has destructive power for micro-organisms quite equal to that of iodoform. (*Lancet*, August 29, 1891.)

Europhen when applied in powder to gingival inflammations, produced by the setting of crowns and bridges, promptly and efficiently reduces the swelling and soreness in two days. When it is applied to mucous patches of the cheek or tongue of syphilitic origin, it is equally efficacious, as I have had abundant opportunity to demonstrate in my own practice.

Applied to a suppurating or an inflamed pulp, it promptly subdues the pain incident to such condition. I apply it in powder or rubbed up with lanolin. In the proportion I use twenty-five parts of europhen to seventy-five parts of lanolin. This is also a very neat way to use europhen in excoriations around the corners of the mouth or the nose. As an agent for inflamed sore mouth under metallic or rubber plates, the lanolin paste is exceedingly desirable. It may be used as an after-dressing in pyorrhœa pockets as long as it is desired to secure the benefits from the liberation of iodine.

Your attention is earnestly recommended to europhen as a parasiticide, where such agents may be required. It is barely possible that it should be extremely useful as a dry dressing in empyæmia of the antrum, but I have not as yet used it for that purpose.

ACIDUM CHLOROCITICUM (TRICHLORACETIC ACID, $C_2HCl_3O_2$).

Trichloracetic acid, discovered in 1838 by Dumas, is made by treating chloral hydrate with three times its volume of nitric acid, and placing the whole mixture in the sunlight until the red fumes disappear. The liquid is then distilled, and the portion coming over at 195° C. is pure trichloracetic acid. The trichloracetic acid is easily soluble in water or alcohol; it is a powerful caustic, quickly destroying the epidermis or mucous membrane. It is found to be in colorless, rhombic, deliquescent crystals, freely soluble in water or alcohol. It is a product of the oxidation of hydrate of chloral by means of nitric acid. It is an escharotic. When diluted with water to three per cent. it is an excellent local astringent and stimulant. When it is used to soften or decalcify the serumal plates attached to the roots of teeth, a ten-per-cent. solution in water should be used. The action of the acid may be nullified with carbonate of soda, magnesia, or any suitable alkaline solution. The trichloracetic acid seems to have that peculiar property of softening calcific and sanguinary deposits on the teeth without injury to the substance of the tooth.

Trichloracetic acid is submitted as an agent of undoubted value in dental therapeutics for the following purposes:

1. Its solvent effect on calculi on the roots of teeth.

2. Its destructive effect on the pus-producing surface of the sockets of teeth.

3. It is a good astringent.

4. It is a stimulant.

5. It is an energetic caustic for the destruction of morbid growth, epulis, and excrescences on the pulps of teeth, overhanging gum on third molar teeth.

6. As a refrigerant mouth-wash in one-half- to one-per-cent. solution in water.

SYRUP OF IRON CHLORIDE.

In all cases where an efficient tonic preparation of iron is to be taken by a patient, the elegant formula of Dr. G. W. Weld is to be preferred to the pharmacopœical preparation so long in use, as it is not injurious to the teeth.

Many members of the medical profession are prescribing Dr. Weld's formula, and it becomes our duty to insist, when iron is to be used internally, on the patient taking the non-alcoholic syrup of iron chloride.

The dose is one tablespoonful, diluted with three times its volume in cold water. It acts as a diuretic in anæmia, erysipelas, diphtheria, and neuralgia, and in many cases it has no equal as a prompt remedial agent.

DISCUSSION.

*Dr. Abbott.*—I am both astonished and pleased. I am astonished that such men as Koch, for instance, should have made a mistake, —astonished that so many men, after his experiments with bichloride of mercury, should have kept on making mistakes. It seems that every year a prop is knocked out as far as antiseptics are concerned. We will soon find that we have nothing that will destroy germ-life, and that germs are as thick as ever, without our possibility of destroying them. If there is anything in the world that will destroy organic life it is bichloride of mercury. It will kill positively. If it will destroy human life and all kinds of animal life, we must naturally suppose that it will destroy the organisms which prey upon human or animal life.

From the paper read by Dr. Hunt, we are led to believe that nothing will destroy the organisms about the mouth. In our practice, however, we have seen wonderful results from bichloride of mercury. It does away with the possibility of suppuration, and a general disturbance brought about by the organisms, and we rely upon it, as all surgeons in the country do. If that be true, it seems that there must somewhere have been a mistake in the work of this last experiment. I do not believe any man is infallible, and sometimes the variation of a hair will give results that will carry him almost entirely away in direct opposition to others; so we have just as much right to continue the use of bichloride of mercury as we ever had, and we can get good results from it just as well, notwithstanding the experiments made.

In the treatment of pyorrhœa alveolaris and all alveolar diseases, there is nothing that will take the place of chloride of zinc. It is, in the first place, an escharotic, a stimulant, and a powerful astringent. We have all three qualifications, and besides this it is quite a strong antiseptic, so that it gives us for certain uses around the mouth qualities that we cannot get from any other substance that I know of. It will not stain anything that you use like sulphuric acid. Sulphuric acid, if it touches your clothes or your napkins, destroys them. If chloride of zinc comes in contact it does not harm.

The remedy that Dr. Harlan has referred particularly to, an agent for treating general diseases of the mouth, I know nothing about, but from his statement I fancy it is a very excellent thing. If it will relieve those painful points under plates it is certainly a remedy that every one should have in his office. If it will relieve the pain around the roots of teeth where crowns have been set, or where tartar has been removed, it certainly is worth much in practice.

The preparation of iron prepared by Dr. Weld, of New York, is somewhat peculiar. It is claimed that it is not an acid, and will not affect the teeth; but there is another feature about it. Let it stand in your office for a week or ten days, and you will get a precipitate one-quarter the depth of the bottle. The iron seems to precipitate, and you get comparatively no iron except when you shake it, and then, of course, it is not in perfect solution. Whether that has been overcome I cannot say; but I think it has. Of course Dr. Weld's preparation is a most valuable one, if what has been said about it be true, because if anything has done injury to teeth it is this chloride of iron. Anæmia always follows that condition of malarial poisoning that we find in malarial districts, and the treatment that is generally prescribed is first quinine, and then the tincture of chloride of iron. It is an excellent preparation as far as the general condition of the system is concerned, and to retain the restorative properties of the preparation that are in the tincture, and still get rid of the bad effect which it has on the teeth, is a wonderful thing to have accomplished, as far as the welfare of the people is concerned.

*Dr. Marshall.*—I want to refer to one remark that Dr. Abbott made in regard to the use of antiseptics. He said that almost all surgeons rely upon bichloride of mercury in their operations. That was a very broad statement. There are many surgeons who do not use it at all, but rely simply upon sterilized water, especially those who make abdominal sections. There is one surgeon in Chicago who has not for the last three years used bichloride of mercury at all. He simply uses sterilized water. His instruments are all boiled before being used. His own hands have been thoroughly cleaned with soap and sterilized water. The parts are washed with soap and water before the operation is made, and then washed off again with the sterilized water; the sponges have all been boiled; the irrigator is charged with sterilized water, and the abdomen is washed out with it. Stitches are applied after the operation, and it is again washed with sterilized water. I have seen many of those cases, as I am connected with the same hospital, and he has had better results by the use of the sterilized water than any of the other men have had with the bichloride of mercury solution.

In regard to the report on anæsthetics, I have never used pental, but just before leaving home I came across a report from Professor Goerck, the chairman of the Committee on Anæsthetics of the German Association, in which he gives a report of 109,230 cases of narcosis, in which 39 proved fatal. Of this number, chloroform was used 94,123 times, with 36 deaths, or one in 2614. That is a little larger than is generally estimated. The general estimates have been one in 23,000 to 25,000 in chloroform. Out of 8432 cases of narcosis with ether, there was one death. That is a better percentage than we used to know about. We used to say one in 5000. There were 2891 cases of anæsthesia with chloroform and ether combined, with one death. There were 1380 cases of ether and alcohol combined, with one death. There were 2179 cases in which bromo-ethyl was used, with no deaths, and 219 in which pental was used, with one death, proving from its report that pental is the most dangerous of all.

*Dr. Peabody.*—There is to my mind no subject in our profession which has as much interest in connection with it as that of this section. Our information in regard to therapeutic agents has been rather limited. We have too many agents altogether in our dental practice, and yet we have too few. New discoveries are being made yearly; old and tried ones are laid aside. The new ones will, in the hands of certain operators, produce results which cause the larger proportion of the profession to rush to it, expecting effects of a similar character. In probably fifty per cent. of the cases where these new remedial agents have been tried, they have been followed by failure. We, of course, want new agents that will produce better results than those we have at present, and we are constantly in search of them. Those gentlemen who are capable of making investigations of this character are the lights to whom we look for information, and we receive it from that source. But occasionally some one who is not gifted with an immense amount of chemical information by accident stumbles on something which produces equally as good results as those which are brought before our profession by men of a greater degree of intelligence. For my part, I hardly understand why old, tried, and true remedial agents are cast aside. We have had good effects from such agents as carbolic acid and creosote, the first things we used. Only a short time ago I was struck by two statements that I saw in dental journals. One person in writing on carbolic acid said, "Creosote

I have no use for." Another said, "Carbolic acid I have no use for." They probably received benefit from the use of other agents, and on that account they were inclined to look down on those with which others had been satisfied. Iodoform has been in the hands of the dental profession for some time. Different men have produced different results. Some depend upon it entirely in connection with the oils, while others have no use for it at all. They prefer something of a different character, partially on account of the disagreeable odor.

I have brought to the Association, for the purpose of allowing the gentlemen to see it, a very simple instrument, composed of a tube passing through a cylinder, the tube being closed from the centre, perforated on each side of the closure for the discharge of gases. This cylinder has been used only with iodoform. The idea is to unscrew this cap, put in some crystals of iodoform, hold it over the flame of the lamp, and placing it in the hands of the patient, request him to use it, and by holding this in the tooth, the vapor of the fused iodoform is passed under pressure into the tissue.

The results of the use of this agent have been wonderful. I cannot enter minutely into the properties of it, but I want to state more particularly the effects that follow its use. By the heating of this cylinder the iodoform is fused and iodine is eliminated. That passes into the tissues, disinfecting them. Iodoform is considered not to be a disinfectant; some say it is not an antiseptic; in the form of vapor it certainly controls septic troubles. The passage of the vapor of iodoform into the canal of the tooth, which is filled with pus, will disinfect the tooth and destroy the pus and inflammatory conditions. So capable is this vapor of iodoform under pressure (I am not speaking in favor of iodoform, because I think it can be superseded by something of a better character) that there is no portion which it cannot reach. For instance, placed in the canal of a tooth, the vapor will permeate every sinuosity, disinfect the tooth thoroughly, and leave behind it a solid, insoluble precipitate of iodoform itself. I have a tooth here which was subjected to the vapor,—the root has been ground down. Even the tubuli are filled with this iodoform precipitate. It reaches the peridental membrane, and as it is a non-irritant and is in the condition of vaporization a disinfectant, irritation is reduced, the septic matter is destroyed, and the tooth, by means of that instrument alone, restored to a normal condition, as far as a tooth which is deprived of the living tissue in the pulp can be restored. Loose teeth have been reduced to a condition of firmness. In the College of Dental Surgery this was in the hands of students who were not experts, and there have been six hundred teeth subjected to this process. Out of that number, as far as is known, there have been but two failures. This process has been in use for about three years, and the person who introduced it states that he has had but one or two cases returned. In one of these failures, in the infirmary, the student having charge of the case subjected an inferior bicuspid tooth to this treatment.

In using iodoform the odor is very objectionable. Under the vapor the odor is not as perceptible as in the ordinary condition. The greatest objection is the fact that it has such a bad effect on the instruments. Upon almost every occasion I have found the instrument covered with a film of iodide of iron. In a very short time it will rust any instrument brought in contact with it. It occurred to me that perhaps aristol might be a substitute for iodoform. Dr. Hunt stated that aristol was a non-irritant. It has been experimented with very slightly with this instrument; not sufficiently, however, to form any absolute conclusion in regard to it. Still, the slight experimentation which it has undergone has led the operator to believe that it was an irritant.

I consider the application of vapor under pressure an idea which is calculated to a great extent to revolutionize the practice of therapeusis in general surgery.

I will state that the ordinary crystals of iodoform will not produce such good results as the conglutinated forms. Whether they are adulterated or not I do not know, but they do not produce as heavy a vapor or as heavy a precipitate. I hope you will investigate this matter, and in the course of the next twelve months come prepared with something that is superior to anything we have.

*Dr. Hunt*, of Indiana.—I want to say a few words in regard to bichloride of mercury. Dr. Abbott very truly stated that it was sure death to animal life, but we know that the higher and more complex the organization the greater the influence of deleterious agents on it, and the lower the organization the more it will stand. For instance, a snake displays muscular activity after its head is chopped off, so does a turtle and a chicken; but as we ascend in the scale of living organisms towards man, the most perfect, we find that immediate death is induced by such catastrophes as cutting off the head. Then, again, while bichloride of mercury may be certain death to animal organisms, it has not been successfully proved that it is always certain destruction to vegetable parasites.

The portion of the report referring to bichloride of mercury was as new to me two weeks ago as it was to you gentlemen this morning, but I have been assured that the experiments were very carefully conducted under the supervision of very competent persons of considerable experience in that line, and I think it probable that there may be more in it than we at present believe. We know that in surgical operations, even with the best antiseptic precautions and the use of bichloride of mercury, we have occasional failures. Every once in a while there is septic poisoning, even with the use of the best antiseptics. When that occurs we are all prone to lay it to the fact that we have used something that has not been thoroughly disinfected; but that is only a supposition; and might we not as well suppose that when we have used bichloride of mercury the conditions have been such as the paper mentions; in other words, that the germs have been covered with an albuminous coating by the bichloride, and that it has been washed off in the blood and sepsis has resulted? It seems to me that that part of the subject requires further investigation before it can be disposed off.

The use of chloride of iron is very much to be commended, and in this connection I would like to state that the use of chloride of iron pills are preferable to any other form of iron when the patient's stomach will digest that. I have in mind a case of anæmia complicated with gastralgia. The patient had had it off and on for ten or twelve years, the attacks being not at all regular, but very severe. I gave her the chloride of iron preparation for about a month, and followed that up with strychnia, one-sixtieth, and then chloride of iron pills, keeping up the treatment for three months, and six months have passed with no return of the gastralgia. So if the patient will stand it, those pills are the best way to administer the chloride of iron.

In regard to Dr. Peabody's suggestion on iodoform vapor, I don't think there is anything particularly new about it. The method of using iodoform vapor in the root-canals was fully discussed by Dr. W. H. Whitslar, of Cleveland, five years ago, in the *Dental Review*, and it has been quite thoroughly proven that while the iodoform vapor is heavily charged with iodine, and is antiseptic, yet the iodine disappears very readily and very quickly, leaving behind only a carbonaceous residue. This is aseptic, and not antiseptic, and therapeutically inert; but it is far better than septic material in the root. The iodine is all evaporated, leaving this material behind, as can be demonstrated by the use of the microscope.

*Dr. Abbott.*—There is one point in reference to the use of antiseptics which it would be well for us to bear in mind. You will remember that many years ago we had the impression that carbolic acid was one of the best antiseptics ever known. I think the authority for this idea came from Germany. One person will state that carbolic acid is the best preparation as an antiseptic; another experiments with it, and he finds that he has seen germs of different kinds working their way around over crystals of carbolic acid, and that they were not at all disturbed by it, but lived near it as well as anywhere else. Investigation showed that carbolic acid, when properly diluted, would destroy germ-life.

In the experiments described in the paper very strong preparations were used. A saturated solution was one in which the germs lived a certain length of time; another was a twenty-per-cent., and another a fifty-per-cent. solution, and so on. Should these solutions be too strong, reduce with water, and the effect may be better. For instance, 1 in 20,000 of bichloride of mercury is a good solution. I use that for washing out canals of teeth and abscesses. I never use anything to throw into the mouth stronger than 1 in 10,000, and it was determined some time ago that a preparation of 1 in 100,000 was strong enough to destroy any kind of germ-life in existence, and this only occurred a short time ago. It seems very curious that so many men should be experimenting with this material and getting such different results. There is something wrong about it; they do not work alike, or they certainly tell what is not true as a result of their experiments.

*Dr. Low*, of Buffalo.—I remember a very interesting series of experiments of Dr. W. D. Miller with bichloride, carbolic acid, iodoform, and many other medicaments, and if his conclusions are correct, the bichloride of mercury seemed to be the only one which would destroy life. I believe the proportion was 1 to 480.

In regard to root treatment, if others had had the same experience with iodoform that I have had they will not use it, on account of its odor and the taste it leaves in the mouth. From some experiments that I have made, I do not believe that it is possible under any circumstances, with any vapor of any substance, to perfectly produce antiseptic conditions in the root; and if we remove what we can with bichloride of mercury, or peroxide of hydrogen, which, by the way, has not been mentioned, and completely dry out those pulp-canals and fill them with an inert substance like chloro-percha or gutta-percha, we can depend upon nature to eliminate the germs there. One case, I remember, was in the mouth of a female patient about thirty years old; it was treated very carelessly with peroxide of hydrogen. In twenty minutes the tooth was filled; the gum was painted with bichloride of mercury, the extracting-forceps used, and the tooth placed in solution. The best microscopist in Buffalo carried on the experiment, and nothing was developed. The same experiment was tried with a patient equally healthy, and the tooth extracted immediately after, and the bacilli were found immediately. So we must rely on nature after all.

*Dr. Fillebrown.*—It is not bacteria that make trouble in the human system. Put bacteria of any kind into the living, healthy tissue, and they will perish as quickly as they will in any of the antiseptics or germicides that you have mentioned here. It is only the dead tissue that forms food for them. When we get blood-poisoning into the system, it is from their products. I am not giving anything new; I am simply calling your attention to facts. If you will perform a surgical operation of any kind, and will be sure to leave no *débris* there, if you will bring the surface close together, I will take all chances for the flourishing of bacteria. I think the use of antiseptics or germicides is very much over-estimated. As Dr. Abbott said, when the use of bichloride of mercury gives such widely-different results in the hands of different men there is something that is not comprehended and not used rightly; there is some fact not yet discovered, and within the last fifteen years the disuse of germicides has been growing, and many of the most eminent surgeons are to-day doing their operations without the antiseptic precautions that Lister first advocated.

It has been proved by a long series of experiments that a solution strong enough to destroy the germs will be strong enough to exceedingly injure the delicate tissues on which we operate. Just bear in mind that thoroughness, carefulness, and cleanliness are of more importance than the antiseptic solution that is used, and that the operation which is clean fulfils the essential principles that are required in all these cases that we have.

*Dr. Peirce.*—I have kept my seat, hoping somebody would say what I had to say. The paper read by Dr. Harlan made reference to the trichloracetic acid. About a year since Dr. Kirk asked me if I had tried it in my practice. It has had such eminently successful results that I must say a word about it. Where you have a spongy condition of the gums which you desire to remove, or a growth over a third molar, or any condition of that kind, it is the most appropriate application that can be made, by simply taking a little wooden spatula, saturating it, and rubbing it over the tissue.

It is a powerful escharotic, and in one or two applications you will remove the abnormal growth. Again, where you have the little nodules of calcific deposit on the roots of the teeth, with the same little wooden spatula you pass down into the apex and cleanse off the surface of these roots. I have no hesitation, wherever we have the calcific deposits, in using this acid, even in its full strength, in that way. It cleans off the root thoroughly. After you have used your scalers, use this, and it will remove every vestige of the calcic deposit. Then, again, it has a very happy result on the tissues themselves. Being a powerful escharotic and astringent, you will find that an application into the pocket will arrest the accumulation of pus that is so common, with one or two applications. I have had very desirable results from its careful use in all the cases of pyorrhœa of this nature that I have treated. I have no hesitation, if I open into the root of a tooth, to dress the pulp by taking a little circular spatula and forcing it up into the root; the acid will destroy the tissue, and you purify that root, in a moment's time, more perfectly than you can with carbolic acid.

*Dr. Patterson.*—The report of the Section is interesting, especially that in reference to nitrate of silver. We all have a great number of young patients, between two and three years of age, whose deciduous teeth are threatened with incipient decay. It is almost impossible to introduce any filling-material in their teeth. It has been suggested that incipient decay can be arrested by the application of nitrate of silver. In that I have been experimenting, and I do not know what the result will be, because the experiments are very recent. If we can arrive at a solution of that difficulty in this case it would be a great thing. I have been told, since this paper has been read, that this is not a new treatment; this practice had been used in former years. If any one has experience in that line I should be pleased to know what the result has been. So far as I have been able to watch it in the three cases in which I have tried it, it is entirely satisfactory. I do not care if it discolors and makes the cavities as black as can be, so long as it preserves the deciduous teeth.

*Dr. Taft.*—In regard to the use of nitrate of silver, I remember that more than forty years ago Professor James Taylor, in his instruction in the Ohio College of Dental Surgery, recommended that treatment for decay of the teeth. Recognizing then the fact that the darker varieties of decay were the slower, the suggestion was made that by the use of the nitrate of silver the decay was changed from the light and rapid to the dark and slow. It was used in the deciduous and permanent teeth, and from that time to the present day I have been in the habit of using this material in certain cases, especially in the deciduous teeth, and I have been warranted in continuing the use of it from the good results received. A year ago, at Saratoga, this subject was presented by Dr. Stebbins, of Massachusetts. He presented a boy whose teeth had been treated, and the decay seemed to be arrested.

I have experimented as much in permanent teeth as in temporary ones, but it has always been attended with good results. As to how the result is produced, my impression is that it is by its antiseptic powers, and by its destruction of the germs that are operating in the process of decay. That it does produce good results, and that it was used as long ago as forty years, I know positively.

*Dr. Truman.*—Ever since I have been in the profession I have attended dental meetings, and whenever any new subject has been presented there has always been some one to claim originality. Dr. Taft says he has used nitrate of silver for forty years. Why has he not given it to the profession? I have no confidence in that form of dentistry. I have been in the profession over forty years, and I think I am tolerably familiar with its literature throughout that period, and I have yet to see a single paper on nitrate of silver that did not take the position that if you use it in teeth it will so discolor them as to be injurious. If Dr. Taylor or Dr. Taft had been in the habit of using this agent for the purpose recommended by Dr. Stebbins at Saratoga last year, they are to be censured for not publishing it. Dr. Stebbins I hold to be the originator of this process, and it is one of the most important things introduced in dentistry of recent years. I am not willing to sit here quietly and have the credit placed where it does not belong.

On the general topics that have been before us I know almost nothing. I have experimented with germicides perhaps as much as the majority, but I do not believe there is a single germicide extant to-day that you can absolutely depend upon. Therefore we must wait for future investigation before anything positive can be determined. Clinical observations do not always support those of the laboratory, and the results in the latter are not always sustained in practice.

*Dr. Taft.*—Either what I stated is true or it is not true. Dr. Truman says it is not true.

*Dr. Truman.*—I deny that I made any such statement; I said simply that it was not given to the profession; and if true, no credit of discovery was deserved.

*Dr. Taft.*—I did not claim originality; I simply stated where I saw it first used, and that I used it since that time myself, and I have not kept it a sealed matter all these years. I have occasionally spoken of it. I do not remember whether I mentioned it in this Association, but I have referred to the source of my information in regard to it, and the results of my own experience. I think the statement Dr. Truman made needs a little modification. I know also that things oftentimes are presented anew; things that have been long ago forgotten are revived or revised and brought forth as a new thing. I know that is true in regard to nitrate of silver.

*Dr. McManus.*—Nitrate of silver has been used for many years. I know my preceptor frequently employed it about the necks of teeth, where they are often sensitive. I know about five years ago a lady came into my office whose teeth were in that condition, and a dentist had been treating her with nitrate of silver. Her teeth had been so sensitive that she could hardly brush them, and after the use of this agent they were very comfortable, the only objection being the discoloration. I know Dr. Stebbins well. He is an old friend of mine. I saw those cases before they were presented at the Saratoga meeting, and I have seen them since, and I know that they have been very successful. They have arrested the decay, and the use of nitrate of silver has made the teeth very comfortable. There are many remedies used by men who have never attended society meetings. The unfortunate thing is that there are many who do not attend these associations. They do not come for many reasons; one of them is, that they are barred out of local societies, do not get into membership, and of course are not appointed as delegates. My old preceptor never attended a society meeting in his life, and as far as general intelligence is concerned he was the equal of many now present.

*Dr. Haroun.*—I have known Dr. Taft intimately since 1858; I knew Dr. James Taylor a short time afterwards, and both of these gentlemen have reported the use of nitrate of silver, I myself having used it through information received from them. It may not have been reported in the journals, but I have heard them speak of it. We knew it would blacken teeth, but used it regardless of discoloration.

*Dr. Barrett.*—I have heard Dr. Taft refer to this repeatedly, and I can place my memory upon the exact time and place for one of those periods: that was at the meeting of the Michigan State Dental Society. I know he has reported it and spoken of it frequently. I

apprehend that he thought it was so well known and was such an old remedy that all that was necessary was to call the attention of those present to it. It is really an accepted remedy in practice, having, however, certain disqualifying properties, and there are certain things in it that are objectionable.

*Dr. Truman.*—I did not implicate Dr. Taft. I simply said, Why did he not publish it to the profession? He has stated it occasionally at conventions, but a series of experiments, such as Dr. Stebbins made, running over six years on one agent, I have yet to see from Dr. Taft's pen or from that of any other person. I am perfectly familiar with the use of nitrate of silver in dentistry for the past fifty years. My memory is not treacherous.

*Dr. Moore.*—I want to call Dr. Truman's attention to the fact that in an institution in which he was professor for a long time, —twenty or more years ago,—we were told to use that remedy not for the purpose of treating pyorrhœa alveolaris, but for sensitive dentine. Professor Truman, Dr. Peirce, and other gentlemen doubtless remember this.

*Dr. Peirce.*—I do not remember the time when I did not keep my stock of nitrate of silver in the office for the purpose of obtunding sensitive dentine, but that is very different from the systematic experiments made by Dr. Stebbins. I have taught that it was one of the best materials we have for relieving the sensitiveness of a tooth where it is to be cut down. I have taught that to every class. It was spoken of twenty years ago, but not for the purpose of arresting decay, and I must coincide with Dr. Truman when he says that Dr. Stebbins was the first one who published a systematic method of using it.

*Dr. Peabody.*—Over twenty years ago I used nitrate of silver on many occasions for the purpose of relieving the sensitiveness of teeth at the necks, and I have known of it since that time; but it is only within the last twelve months that I ever heard of it for arresting decay. Dr. Taft said he had heard of it for the purpose of changing light decay to dark decay, because dark decay progresses more slowly. I never heard of it in that way, and its use by the profession, as far as I know, has been for the purpose of relieving the sensitiveness, until Dr. Stebbins introduced the idea of arresting decay.

*Dr. Crawford.*—I do not expect to be able to throw any additional light on this question. There is no doubt to my mind but that nitrate of silver has been recognized as an agent in the treatment of diseases of the oral cavity ever since the organization of the dental profession. These gentlemen are both right; both sides are right, and yet both are wrong. The only point at issue is as to who is entitled to the credit for the application of nitrate of silver for the treatment of children's teeth. Dr. Stebbins is entitled to that honor; but that Drs. Taylor, Taft, Murray, and Harris, and all men who have written works on the subject of dentistry, referred to nitrate of silver, is certain. However, none have made the high claim that it was capable of arresting carious action. Most dentists dwell upon the idea of using it as a comfort-giving application, just as they use it as an application for a sore or ulcer in the mouth. That is a synopsis of the history of nitrate of silver, as far as I know.

*Dr. Harlan.*—The points brought out in the discussion are rather satisfactory to the committee making the report, but to clear up the cloudiness surrounding the subject of the use of bichloride of mercury solutions, I would state that the experiments of Laplace, of Philadelphia, showing five parts of tartaric or hydrochloric or carbolic acid added to the 1 to 1000 solution of bichloride would make it absolutely reliable as a destroyer of micro-organisms. There have been no experiments anywhere to disprove that. That conclusion has been arrived at clinically by Dr. Black, of Jacksonville, who dissolved his bichloride of mercury in peroxide of hydrogen. Dr. Black has never had reason to abandon the use of it on account of its failure to destroy micro-organisms in the human mouth. The general subjects treated of by the secretary of this Section have been very well discussed, and he has made a very excellent *résumé* of what he had to say. The point that I desired most discussed, with reference to the properties or use of europhen and trichloracetic acid, has been lost sight of, except by Professor Peirce. For the benefit of those gentlemen who did not hear the paper, and who are going to make renewed efforts, I will say that europhen is an agent that is destined to take the place of iodoform; it is almost non-odorous, non-poisonous, five times lighter, and will answer admirably for all the purposes for which iodoform has been used. The trichloracetic acid, as has been stated, is very useful for the solution of those particles of sanguinary deposits on the roots of teeth that are almost inacessible. It is useful as a stimulant and astringent, and to check the formation of pus on the mucous membrane of the mouth, and it is a very excellent refrigerant mouth-wash in all inflamed conditions of the mucous membrane. As a mouth-wash, use one-half to one per cent. in water. It is freely soluble in water, and for the removal of deposits on the roots of teeth a ten-per-cent. solution in water may be used, and it may be followed by magnesia, sodium, silicate, or any solution that you may have handy.

Subject passed.

Section IV.

Dr. Abbott, chairman.

*Dr. Abbott.*—I have been trying for months past to have an instrument made to show you for the purpose of illustrating certain work. It is simply a camera for making micro-photographs which we could not procure. I expect with it to produce very excellent results, and which will aid in clearing up some points still in doubt.

I have only one paper, and it is a voluntary article, and with your permission the writer will read it. It is one of the most interesting cases I have ever seen, and I think it will strike most of you as being very peculiar.

Dr. Stainton, of Buffalo, then read the paper described.

### CROWNLESS TEETH.

BY C. W. STAINTON, D.D.S.

It has been my fortune to have in my practice a family presenting some traces of heredity rather unusual in their character.

I exhibit to you casts of the mouths of three members of this family, being one-half of the children in it. The peculiar hereditary strain is the absence of crowns to the teeth.

The history of the condition is, as usual, quite meagre. The father is an intelligent man of business, one of the prominent men in a large city, of good physical organization, having six children by two marriages. By the first marriage he had three sons. The eldest has splendid teeth; the second and third sons are specimens of this peculiar condition, and I exhibit the casts of their mouths marked 1 and 2.

By the second mother there were two daughters and one son. The eldest by this second marriage, a young lady of eighteen, is the case presented in cast No. 3.

The history is unsatisfactory from a professional stand-point, but, as Dr. Talbot says, "as good as one ever gets" is about like this. The maternal grandmother had such teeth; she inherited it. Back of that the line is broken. Whether any of her brothers or sisters had such teeth is unknown.

The only certain point known by the father is that he inherited this from his mother's side, and that she also inherited it. His mother had eight children; his teeth were the most defective. Two others had very poor teeth; but whether they were wholly crownless, like his, is uncertain. His idea is that four of the eight had very good teeth, one middling, and three poor. That is all the history I can give you.

When I first became acquainted with the father, about twenty years since, he was wearing an upper denture, which articulated against a few scattered roots in his lower jaw. I have made him in that time two full dentures, opening his mouth probably three-eighths of an inch, improving greatly the contour of his face by removing the too near proximity of the nose and chin. This peculiarity, the shortening of the facial contour, the causation of which will be apparent to all, is the chief distinctive feature or deformity in all these cases. It is especially apparent in the third case, where the well-developed miss of eighteen years, in consequence of the shortening of the face, has a retreating chin. (See model No. 3.)

The first case, that of the second son, came under my observation in 1879. He had occasion to visit me several times on account of the roots becoming sore and troublesome. I removed two at different periods for this reason. In the summer of 1881, he having reached eighteen years of age, and his mouth apparently being well developed, I extracted the roots in the upper maxilla, and in September following made him a set of teeth. He had been waiting with great impatience for this time to come. It was almost impossible for him to articulate satisfactorily, and his course in elocution at the normal school had been largely a failure, because the tongue had no front teeth to strike against to produce certain sounds.

His personal appearance was greatly benefited; the shortened face became at once nearly normal, and the improvement in speaking and singing was very marked.

I do not think there has been any arrest of development of the arch in this case, although that is an assumption and not a matter of positive knowledge. He is not wearing the original plate; a fit of nausea in April, 1889, ended in its destruction.

He claims, however, that it fitted all right. You will see when you look at the model of this mouth, No. 1, a most remarkable antagonism. Two surfaces could hardly be in more perfect apposition. He could almost bite a thread anywhere about the ridge.

The roots extracted in this case were very imperfectly developed and were loosely attached to their sockets, so much so that I removed them all at one sitting without an anæsthetic and without any complaint on the part of the patient, many being similar to loose deciduous teeth.

The third son, seeing the improvement in his brother's condition, was desirous of some such treatment, but I kept him waiting until he was eighteen years of age. In October, 1885, I made him an upper plate. The model shown of this case, No. 2, is not so perfect a representation of the conditions as it ought to have been. I forgot to take an impression of his teeth until after I had extracted several of the roots.

With a remembrance of the ease with which I had extracted the roots in the first case, I tried to extract these, with the assurance that it would not be very tedious to the patient; but I found they were better developed and very adherent, so that it was extremely difficult to extract them at all; and after removing the roots of the incisors and the bicuspids of one side, I ground off those remaining and made a plate over them.

The model in this case shows the condition after the mouth was prepared for a plate, and not, as it should, before anything was done. A peculiar condition in this case was that the front roots could not be made to touch each other. Of course my model makes this condition more pronounced, because the roots of the incisors are absent and some absorption had taken place in the few months between extraction and the taking of the impression, and I have also smoothed off the roots of the cuspids; but he never was able to get the incisors within a line or two of each other, and yet you can see how smooth the lower roots have become, though these have not been touched by the wheel.

The making of a denture for this case was not nearly so easy as for his brother; to open the bite sufficiently for anything between the molars made the incisors too prominent for a mouth never showing any, and with a lip that lifted as far as the nose would allow at the slightest provocation.

My first attempt was not satisfactory, but a second was fairly successful. This patient is one of those who will crack nuts and bite hard candy with his teeth, so that I have had to replace this plate, made in 1885, simply because he had fractured these thin, pinless molars.

Model No. 1 shows apparently purely mechanical abrasion. Model No. 2 shows the same condition posteriorly; but the anterior roots show chemical rather than mechanical abrasion. This subject tells me he never has been able to bite anything with his front teeth, as they never came together.

Model No. 3, that of the miss of eighteen years, exhibits what neither of the others shows, a fairly good crown to each central incisor. This model is articulated with her mouth at rest; but she has a rather unusual ability to project the lower jaw. This case seems to demonstrate that there was an effort to produce crowns, but so poorly were they organized that they have been easily obliterated. What is left of the centrals is because she cannot protect the lower jaw sufficiently to wear these away.

This model shows plainly another thing, the presence of decay among these teeth. The first permanent molars above, and the first and second permanent molars on one side below, have extensive caries; there is almost no trace of decay in the other cases.

There has been no attempt to do anything in this case. The two central incisors give some appearance of teeth, although they are quite discolored. The young lady assures me that she intends to let them go as they are as long as she lives.

In all these mouths the color of these roots runs from a dark amber to that of an old clay-pipe black with nicotine. I have known all these cases since they began to erupt their permanent teeth. Their teeth have always presented this same crownless appearance; but I am persuaded that the effort was made to give them normal crowns, but they were so soft that they were soon worn smooth.

The lower wisdom-teeth in Numbers 1 and 2, as also several of the teeth in No. 3, are indicative of this. Case No. 1 has apparently transmitted this trait to his children. A little son about four years old has crowns to the deciduous teeth so poor that they dissolved almost as soon as they appeared through the gums.

Of course, we can only conjecture what the permanent teeth will be, but the father has no faith that they will be any better than his own, and I think he has reason to fear this result.

DISCUSSION.

*Dr. Abbott.*—The history of these cases carries us back to embryology, to the formation of these teeth. The question as to how it occurred and what produced it is something that we will be unable to determine, but that the enamel organ has been placed in a condition unfit to perform the proper deposition of lime-salts is clear. We find isolated cases of this kind, where every tooth in the mouth is in the same condition as these. It is a case of improper calcification of the enamel of the teeth, the enamel organ not performing its function. What arrested the function of the organ is unknown. In almost every case it is due to some local disease in the mouth, not to any disturbance, unless it be a very long-continued one. In all the cases noticed of the partial or the general imperfection of the enamel, it is found to be due to a local disturbance of the mucous membrane. I want that point noted, because I desire you should observe it. In studying the subject microscopically, I have come to the conclusion that no faulty development of enamel takes place, as a rule, where the local disturbance is not present. A little boy came to me some years ago whose front teeth had no enamel at all, and were so stunted that the dentine was but one-half of its normal thickness. I asked the mother if the child had had any disease, and she said no ; the child had been well from the time of its birth up to the time I saw it. The father was a physician, and I told him the child had a very peculiar condition of the teeth, and the mother had stated the child had never been ill. I said some disturbance must have occurred to affect those teeth. He confirmed the statement of the mother, and said the child had always been well. A few days afterwards I saw the mother again, and she said she remembered that the child had some sort of an eruption upon its body, which extended over its head and into its mouth, and the mouth became excessively sore. It did not make the child ill, and, therefore, it was not specially noticed. Here was a local trouble which caused the non-performance of the function of the enamel organ. It stopped its work, and no enamel was produced upon those teeth. Not long ago I had a patient with a set of teeth of a dark-yellow color and very highly pigmented, which indicated that they were improperly calcified. The lime-salts were not packed together in a solid manner, and the organic substance was in excess of its normal quantity, and the result was a discoloration of the teeth. These are discolored in the same way, due to a lack of lime-salts. To get at the cause would be almost impossible. It may have been thrush or some disturbance of the mucous membrane. There are local disturbances which occur on the face, and which often occur in a child's mouth, affecting its gums and the enamel. When a lesion occurs on the mucous membrane it will affect the organ itself. It must be a continued and severe illness, one which stops the growth of the nails and the hair, in order to affect the growth of the enamel of the teeth. It is a most interesting case. I know very well that this same condition occurs in cases where there is apparently good development of the teeth originally. I remember, as a boy, seeing a gentleman who had all his teeth, as far back as you could see, worn down even with the gum, upper and lower, and they just came together as in the cases related by Dr. Stainton.

*Dr. Peirce.*—I should dissent from Dr. Abbott in stating that it was a local result. As far as the history of this case gives us any information, it seems to be a reversion from the grandmother on the father's side. The father's teeth, it seems, were not like them.

*Dr. Barrett.*—The father had exactly the same thing.

*Dr. Peirce.*—It seems to have descended from the grandmother. We have all met with cases similar in character. I remember that of one of my own relatives, where the mother and father were both consumptive. All through the period of gestation the mother was on the verge of the grave. The child was born, and the deciduous teeth were deficient in crowns. The permanent teeth came through, —that is, the first molar, incisors, and bicuspids,—and then were entirely deficient in crowns. The child died with scarlet fever at the age of twelve, but there was never any enamel developed on the deciduous or permanent teeth. It certainly was the result of improper nutrition on the part of the mother during the period of gestation and lactation, and of the inability of the boy to develop any strength during his life.

*Dr. Morgan.*—The case presented is evidently inherited. I rise to say that conditions very similar to this occur from local causes, and not from general disturbances. The teeth belong to the dermoid system. I hold that if the child suffers with scarlatina, chicken-pox, measles, or small-pox at the time the enamel of the teeth is being formed, that the enamel organ is of necessity involved, because it belongs to the general system, and it fails to perform its functions properly. In some cases you find the teeth eroded and little pits on the surface of the teeth, showing that the injury was done to the teeth at the same time, but in different localities, because of the stages of development which existed at the period of the disease, so that you will find the pits on the canine teeth close to the point, on the lateral incisors a little farther up, and on the central incisors much higher up, and so on. If you are careful in making your calculation you can come within a few months of the time when the patient suffered with some of these diseases of the dermoid tissues. We have that disturbance from local causes, and may have this pigmentation as the result of imperfect formation in the membrane which secretes the enamel. I have had the care of children, and I have looked forward with fear, with the expectation of finding the permanent teeth in that immediate vicinity affected by the local trouble. I have had my fears verified by finding that after the teeth were erupted there were spots upon them that soon became discolored and afterwards disintegrated, because of the lack of strength and union which held together the particles. I hold to this as a general principle: that you may have it as the result of general disturbance and also from local troubles.

*Dr. Hunt.*—I simply desire to ask a question about the mouth itself. Everything here, apparently, is fully developed except the enamel organ, and that does not seem to have been entirely inefficient. The writer of the paper gave a history, but did not describe the character of the teeth themselves. I notice that these teeth look as though they were in the process of development and eruption, not disturbed in any way. It all seems to be normal. Here is a portion extending below the gingival margin, and I want to ask whether there was any enamel there.

*Dr. Stainton.*—It presents the appearance of secondary dentine.

*Dr. Hunt.*—Model No. 1 looks as if at some time the cutting-edges, or grinding-edges, of these teeth had been longer. Dr. Abbott's statement was that the enamel organ had not been able to perform its function. It appears to me as if there had been the full function up to a certain point, and then outside influences played a part in removing these lime-salts before the teeth were erupted.

*Dr. Abbott.*—As far as these particular cases are concerned, and that of many others, I have no doubt that there may be something in the way of heredity. As Dr. Morgan stated, there are many things inherited from several generations. The sins of the father are visited on the child, and sometimes it occurs that the sins are visited generations afterwards. It crops out in a portion of the family where we least expect it. I want it distinctly understood that I do not claim that every child that has scarlet fever, whooping-cough, measles, or even small-pox, has defective teeth. If this were the case, we would be a mass of people with defective enamel. The reason is that the disease does not take hold of the mucous membrane locally, or in contact with the point over which the teeth are forming. The enamel organ is not diseased nor affected by these disturbances. It is well known that in scarlet fever there is more or less sore throat. In some cases that becomes intense. The whole mucous membrane is involved, and there is difficulty in saving the child. In those cases the enamel becomes affected. Where measles is expected, the physician first examines the throat to see if there is any disturbance there. If there is, he says the child will have measles. If that disturbance is sufficient to affect the mucous membrane, there will be defective enamel. I do not believe there is a case of one in a thousand where the mucous membrane has not been involved. From a general disturbance, as I have said, that affects every growth of the system, such as the nails and the hair, you may expect defective enamel.

*Dr. Carroll.*—Dr. Abbott endeavored to prove a line of heredity, stating that the sins of the father should be visited on the child, etc. It reminds me of another part of the Scripture in which a man was blind to a great extent, and they asked the question, "Who hath sinned, this man or his father?" You remember the reply, "Neither has this man sinned nor his father." The opinion prevailed then, and does to-day, and is promulgated by Dr. Abbott, that we can inherit disease. As representing a specialty of the great healing profession, I desire that Dr. Abbott and the rest of us be accurate in our statements. I think it is generally conceded on the part of astute pathologists, as well as physiologists, that we cannot inherit disease, that we can inherit only a tendency to it. That was nicely put by Dr. Morgan when he attempted to account for local disturbance. Your mother and father may have died with consumption, but you may not inherit it. You cannot inherit syphilis. You can impart it, but you cannot inherit it. Hence, in treating this subject, we should speak merely of an inherited tendency, which, under favorable conditions, would culminate in a disease. Just as in this case, because the grandfather had this form of teeth, and it was found in the grandson, it should not be called a disease. It is merely a lack of performance of function. The enamel organ that would have brought forth perfect enamel was weak, and, under favorable circumstances, would fail to produce perfect enamel. The mother before childbirth may have been in a weakened condition, affecting the enamel. The child, after birth, may have had the diseases of childhood. This inheritance of disease is a false idea, and we want to be relieved from it. Place the child under favorable circumstances, and, although both parents died of a disease, it will develop strength. Place it under unfavorable circumstances and it will become diseased, and then it would be asserted it inherited it.

*Dr. Barrett.*—If it be a fact that disease cannot be inherited, that we inherit only tendency, then it would be true that in syphilis we only possess the tendency to acquire it. The pitted enamel, furrowed enamel, imperfect enamel, I cannot concede to be wholly due to local or general disturbance. If it be true that it is due to general systemic conditions, whenever there is such a pathological state as shall have produced it in one case, things being equal, it it will always produce it.

Many of us know of cases of scarlet fever which have not resulted in defective enamel. There are many cases in which there have been very severe diseases, and yet the teeth themselves have been perfect. On the other hand, there have been cases that, so far as we may trace the history of them, have never shown any eruptive diseases whatever, yet the enamel was imperfect. How can we account for these things. If a law be a law, it must be unvarying under all circumstances. If it be due to disease in one case, it is always due to it under the same circumstances, and will always produce the same result. Ask almost any mother whether there was not, about a certain time, some form of eruptive disease of her child, and you get an affirmative answer. Nearly all children have them, but they are usually of so light a character as not to produce any serious disturbance. The effect produced, according to what we have heard, would be imperfect enamel from the whole cap of the tooth extending down as far as the development had taken place. I cannot concede that that is a fact. We have not reached the limit of our knowledge in this direction. There is something more which we do not comprehend, but I do not think it can be produced by any temporary change in the condition of the patient. In some cases you find the whole of the enamel down to a certain point entirely defective, but in others there is simply a line across the teeth, a defective spot which may exist on one side of a tooth. If it extended clear about the tooth, that would be additional testimony, but generally there is only a pit. This arises more from defective enamel, and not from constitutional disturbance, and not from local irritation. I believe it is due to a defect in the enamel organ by which it does not perform its functions perfectly, and so it may be that we have a defective point in the enamel, or the whole enamel organ may be defective. There may be a loss of the function, and you have no enamel at all.

*Dr. Morrison.*—The effort seems to be to locate the responsibility of this thing. What is needed is to find out how to overcome the difficulty. In nearly all of these cases the roots are well developed and well secured in the jaw, and, when that is the case, I regard it as a great mistake to destroy such foundations and put in any manner of artificial appliance, I don't care how well it may be constructed. The proper remedy would be to furnish what Nature has failed to do, and that is, put a good metallic enamel on those teeth. I would suggest lengthening the bite a quarter or an eighth of an inch, and putting metallic shells on those teeth. The position of the young lady mentioned in the essay was decidedly a very wise one in not wishing the operation performed.

*Dr. Patrick.*—I think it is a well-established fact that a tooth is derived from the same source as that of the whole organism, and that is the mucous membrane. The dental follicle is brought into existence in the same way, and all its processes developed there. There is nothing better established in histology than that an organism, in the course of its development, may be turned aside, either in whole or in part. The result is there are no two alike. If I had a million before me there would be no two human organisms exactly alike. That growth is affected directly and correlatively there is no question. The enamel of a tooth could not, from the very nature of the case, be affected in its early stage, when it is in a plastic condition. When the enamel is formed it may decay. It may be injured mechanically, but it has no power of reparation. I know of one case in which the deciduous teeth, where they appeared through the gum, were denuded of enamel, granulated, and pitted, etc. That could only have taken place during gestation. The succeeding teeth of that child were perfect. In two other children of the same family the second teeth were defective and the first teeth were not. I have the casts of them, and I know that during the formative period of the enamel, or about that time, they were afflicted with eruptive diseases, and I know that the mother was afflicted with an eruptive disease,—scarlet fever. One of the children had what is commonly called chicken-pox. The other had scarlet fever, and the second permanent teeth were affected.

*Dr. Head.*—Was there anything extraordinary about the mucous membrane of the entire family, either of the father, the mother, or the children? Because, since the enamel organ is formed from the mucous membrane, it might be possible to trace the defect in the enamel organ from some extraordinary appearance in the mucous membrane. During this discussion concerning the cause of deficient enamel in children I was reminded of a case that was directly in our family. My nephew, at the age of three years, was taken with capillary bronchitis and was very ill. The whole system was so overcome that he hung between life and death for a week and a half; and when the central and lateral incisors came through there was a distinct band all around them, where the enamel was entirely absent. In fact, all the teeth which were in process of formation at that time were so badly affected that the boy was really disfigured, and decay took place immediately. But now that the centrals and laterals have disappeared, the permanent incisors that have taken the place of the first teeth have come in with perfectly natural and healthy enamel.

*Dr. Guilford.*—In looking at the models, it occurs to me that this is simply an exaggerated case of a comparatively common condition, and, if I understood the essayist correctly, he was under the impression that no crowns had ever appeared or existed on the roots of these teeth. I think the crowns must have been there originally, although poorly formed, and eventually disappeared by wear. There was probably no enamel on them. The evidences of attrition are plainly visible. In model No. 1 the lower third molar seemed to have the cuspids fairly well developed. The third molar above has not been erupted, and there has been no strain to bear upon them. All the rest are worn smooth. The individual was compelled to eat upon them, and, biting upon the soft dentine, they wore away rapidly. Nature can vary her actions under certain conditions, and, although we do not always understand these, they are nevertheless interesting objects to study. There was a case similar to this presented by Dr. Barrett before the State Society at Williamsport, Pennsylvania. I think it was very much like this.

*Dr. Barrett.*—It was the same case, or at least one of the cases, presented here to-day.

*Dr. Guilford.*—The similarity was so great that I wondered whether two cases of that kind could occur in the same neighborhood.

*Dr. Stainton.*—With regard to the mucous membrane, in only one case was there any difference from a healthy membrane. The mucous membrane looked like the white of a boiled egg. The others were perfectly normal. If any one has looked at case No. 3, and noticed the wisdom teeth, it will be seen that there were no cusps. I have known the young man for about twenty years. He is now about thirty years of age. He has always presented this same appearance of short, ground-off teeth; he never had any toothache, and never suffered with them at all. The objection made by Dr. Morrison, I expected. I had twelve teeth, and I supposed they were inclosed in model No. 1. If the doctor saw those he would say at once that the question of crowning, or a bridge, was entirely out of the question. All the surface they had was the articulating surface, no nerve-canals whatever. Some of you may be able to make a bridge and crown, but when you look at this case you will find that you cannot do it very well. There was not a normally-shaped root in any of them. They looked like fused glass.

The young man came to me when he was about eighteen years of age, and he was very sensitive about his personal appearance. He could not speak well, and I was anxious to help him. He has worn the plate with great ease and satisfaction. The second case was much more difficult, and has not been so successful.

*Dr. Morrison.*—I referred principally to model No. 3.

*Dr. Stainton.*—The young lady does not care much about it. She was pleased with the improvement, although the teeth were as black as could be.

Subject passed.

Dr. Jackson reported, on behalf of the Committee on Credentials, giving the names of the colleges and societies sending delegates, and also the names of the delegates.

Dr. Jackson also reported, on behalf of the Auditing Committee, that the different accounts have been examined and found correct.

Dr. Barrett reported on behalf of the Committee on Amendments, and handed in the report to be printed with the regular transactions.

*Dr. Harlan.*—I have a resolution to offer which has been handed to me. It is as follows:

*Resolved*, That at all future meetings of this Association no dental exhibits of any kind be allowed near the place of meeting."

Carried.

Adjourned.

(To be continued.)

